# Determinants of multiple partner fertility among males in Uganda: a cross-sectional study

**DOI:** 10.1186/s12889-021-11505-1

**Published:** 2021-07-28

**Authors:** Douglas Andabati Candia, Ephraim Kisangala

**Affiliations:** 1 Department of Planning and Applied Statistics, School of Statistics and Planning, Makerere University , Kampala, Uganda; 2 Kairos Hospital, Mukono , Uganda

**Keywords:** Uganda, Multiple partner fertility, Male

## Abstract

**Background:**

Multiple-partner fertility is a relatively new area of study, especially in Sub-Saharan Africa. This study focused on identifying determinants of multiple partner fertility among males in Uganda.

**Method:**

The assessment was carried out using a logistic regression model and secondary data from the 2016 Uganda Demographic and Health Survey.

**Results:**

Among the males, 42% had children with multiple partners. Older age, being Muslim, and being divorced or separated increased the likelihood of multiple partner fertility whereas residing in the Western region, reporting an age at first sex above 19 years and being married or cohabiting reduced the likelihood. Increase in number of wives or partners and lifetime sex partners resulted into a higher likelihood of multiple partner fertility.

**Conclusion:**

There is need to come up with policies and programs aimed at increasing the age at first sex so as to reduce the likelihood of multiple partner fertility among males in Uganda. Government and other stakeholders such as cultural and religious institutions should sensitize and educate the masses on the negative outcomes of having children with multiple partners and promote fidelity for those in marriage. There is also need to increase modern contraceptive use and coverage.

## Background

Multiple-partner fertility is a relatively new area of study especially in Africa. This can be attributed to data limitations given that studying multiple partner fertility entails obtaining relationship data for all births which isn’t normally obtained when conducting fertility studies [1]. These studies usually opt to collect and use data pertaining to marital or cohabiting relationships which makes it hard to identify partners for births that take place beyond the confines of these relationships [1]. Majority of studies regarding multiple partner fertility including Guzzo & Furstenberg [1] and Logan et al. [2] among others have focused on Europe and the USA but few studies have focused on Sub-Saharan Africa [3]. But lately, multiple-partner fertility is being recognized as a problem of perhaps equal importance as non-marital and unintended childbearing as well as childbearing among women below 18 years [4].

Multiple-partner fertility refers to the pattern of a man or a woman having biological children with more than one partner [5, 6]. Previously, multiple partner fertility generally occurred as a result of widowhood and remarriage [2] but at present, divorce and childbearing outside marriage are the main contributing factors [1]. This has potentially negative implications for men, women, and children [2]. These include low quality relationships and increased conflict in relationships where the father or mother has had children from past relationships [5, 7]. Furthermore, having children with multiple partners decreases a person’s chances of getting married especially for women [7, 8]. This is because the society perceives re-marriage among women as a disgrace and abnormal while men are not expected to stay alone thus frequently being persuaded to find another, often younger partner [9]. Additionally, it is difficult for men who have children with multiple women, to cater equally to both financial and social needs of all the families with children from previous relationships usually losing out since more resources are devoted to the present relationship [10, 11].

Besides the negative social and economic implications of multiple-partner fertility, there are health concerns which arise since to an extent, it is a form of risky sexual behaviour. This arises from having unprotected sex with different sexual partners which comes with risks including contracting sexually transmitted diseases. Of the estimated 6000 new HIV infections occurring daily worldwide, two out of three are among young girls and women from Sub-Saharan Africa who bear a higher burden compared to their male counterparts with females aged 15–24 years reporting up to eight fold higher rates of infection [12]. Several studies suggest that men’s multiple sexual partnerships are critical to the spread of HIV in Sub-Saharan Africa with each infected man on average transmitting HIV to 1.5 women whereas each infected woman infects only 0.67 men [3, 13]. Multiple partnerships are closely associated with gender customs of manhood that require men to have multiple sexual partnerships simultaneously to evidence their masculinity [13]. According to the UDHS report, women and men had on average 2.3 and 6.3 lifetime sexual partners [14]. Still, 2% and 21% of women and men respectively reported having more than one sexual partner in the past 12 months [14]. In order to come up with effective interventions to reduce multiple partner fertility among men in Sub-Saharan Africa, policies should be informed by an understanding of the determinants of men’s multiple sexual partnerships which eventually translate into multiple partner fertility.

### Aim

The aim of this paper was to identify the determinants of multiple partner fertility among males in Uganda.

## Methods

### Data

The data used in this study were from the 2016 Uganda Demographic and Health Survey (UDHS). The sample was stratified and selected in two stages. Firstly, 697 enumeration areas (EAs) were selected from the 2014 Uganda National Population and Housing Census (NPHC) comprising 162 EAs in urban areas and 535 in rural areas though one cluster from the Acholi sub region was eliminated due to land disputes [14]. At the second stage of sampling, households were selected. A listing of households was compiled in each of the 696 accessible selected EAs from April to October 2016. Every EA that was selected and had more than 300 households was segmented and one segment selected for the survey with probability proportional to segment size and it’s within these that household listing was conducted [14]. Therefore, a 2016 UDHS cluster was either an EA or a segment of an EA. In total, a representative sample of 20,880 households (30 per EA or EA segment) was randomly selected [14]. The allocation of the sample EAs featured a power allocation with a small adjustment because a proportional allocation would not have met the minimum number of clusters per survey domain required for a DHS survey [14]. The sample EAs were selected independently from each stratum using probability proportional to size. Therefore, the final sample size considered for the survey was 16,206 households. A specific questionnaire was administered to all males aged 15–54 years [14]. The sample of 5336 males was reduced to 3206 respondents omitting all males who had not fathered any child.

### Statistical analysis

Data were analyzed using STATA Version 14.2 at three stages. Firstly, a descriptive summary of all probable independent factors was done. For categorical variables, frequencies and percentages were used. For non-categorical variables, the mean and standard deviation were used. Secondly, using the Pearson’s chi-square test, association was tested between the multiple partner fertility (MPF) status of a male and the plausible independent factors that were categorical. For the non-categorical independent variables, simple logistic regression was used. Independent variables that reported a significant association (*p* ≤ 0.05) with the MPF status of a male were considered for further analysis. Finally, a logistic regression model was run to identify the significant determinants of a males’ MPF status. This was appropriate given that the outcome variable was binary since it was composed of two possible outcomes i.e. a male having fathered children with one partner or having fathered children with multiple partners. The dependent variable was reconstructed from men’s responses when asked about the number of women they had fathered children with. The responses originally included zero, one and more than one woman. Those who responded with zero children were dropped since they had not fathered children with any woman.

## Results

Table 1 presents a description of the respondents. The highest proportion of males had fathered children with one partner (58%) with only 42% reporting to have fathered children with multiple partners. The highest proportion of males was aged 30–34 years (21.1%) followed by those above 44 years (20.1%). The majority of respondents resided in rural areas (80.3%) with the highest proportion in the Eastern region (28.4%) of Uganda. The highest proportion of the males had attained at most primary education (58.4%); were married (61.8%); Catholic (40.7%) and in the poorest wealth index (21.5%). The highest proportion of the males: had their first sexual encounter aged 18 to 19 years (28.1%); used no contraceptive method (55.9%); were not circumcised (57.1%) and had no sex partner other than their spouse (77.6%). The males had an average of one wife with a standard deviation of 0.57. On average, the males had 9 lifetime sex partners with a standard deviation of 17.36.

**Table 1:** Characteristics of the study participants

Variables	Frequency	Percent
Fertility status		
Single partner fertility	1859	58.0
Multiple partner fertility	1347	42.0
Age		
Below 25	366	11.4
25–29	559	17.4
30–34	676	21.1
35–39	476	14.9
40–44	485	15.1
45 plus	644	20.1
Region		
Central	709	22.1
Eastern	911	28.4
Northern	747	23.3
Western	839	26.2
Residence		
Urban	633	19.7
Rural	2573	80.3
Education level		
No education	183	5.7
Primary	1873	58.4
Secondary	745	23.2
Higher	405	12.6
Religion		
Anglican	1141	35.6
Catholic	1304	40.7
Muslim	385	12.0
Pentecostal	286	8.9
Others	90	2.8
Marital status		
Never in union	87	2.7
Married	1981	61.8
Cohabiting	882	27.5
Widowed	21	0.7
Divorced/Separated	235	7.3
Wealth index		
Poorest	688	21.5
Poorer	647	20.2
Middle	650	20.3
Richer	634	19.8
Richest	587	18.3
Age at first sex		
Below 16	646	20.2
16–17	783	24.4
18–19	901	28.1
20–21	419	13.1
22 plus	457	14.3
Contraceptive type		
No method	1792	55.9
Traditional method	161	5.0
Modern method	1253	39.1
Circumcision status		
No	1831	57.1
Yes	1375	42.9
Sex partners excluding spouse		
None	2487	77.6
One	594	18.5
Two plus	125	3.9
	Mean	Std. Dev
Nos. of wives/partners	1.06	0.57
Nos. of lifetime sex partners	9.43	17.36

Table 2 provides a summary of the results of associations between MPF status and the plausible independent variables. Only age, region, religion, marital status, age at first sex, number of wives/partners a male had and number of lifetime sex partners had a significant association (*p* ≤ 0.05) with their MPF status. Males aged 40–44 years (55.1%) had the highest proportion fathering children with multiple partners with the probability generally increasing with age. Regarding the region where one resided, the Eastern region (45%) had the highest proportion of males fathering children with multiple partners whereas the Western region (64.7%) had the highest proportion of males fathering children with one partner. Regarding religion, Muslims (53%) had the highest proportion fathering children with multiple partners followed by Anglicans (41.6%) and Catholics (41.5%). As regards marital status, cohabiting males had the highest proportion fathering children with multiple partners followed by the married (44.7%). Furthermore, the highest proportion of males who fathered children with multiple partners reported having sex for the first time aged 16–17 years (47.8%) and this generally reduced with increase in age of first sex encounter.

**Table 2:** Relationship between multiple partner fertility and selected variables

Variables	Fertility status		n	*p*-value
	Single partner	Multiple partner		
Age				
Below 25	82.5	17.5	366	0.000
25–29	68.2	31.8	559	
30–34	58.6	41.4	676	
35–39	49.2	50.8	476	
40–44	45.0	55.1	485	
45 plus	50.9	49.1	644	
Region				
Central	55.9	44.2	709	0.000
Eastern	55.0	45.0	911	
Northern	56.1	43.9	747	
Western	64.7	35.3	839	
Residence				
Urban	59.7	40.3	633	0.325
Rural	57.6	42.4	2573	
Education level				
No education	56.3	43.7	183	0.127
Primary	56.8	43.3	1873	
Secondary	58.8	41.2	745	
Higher	63.0	37.0	405	
Religion				
Anglican	58.4	41.6	1141	0.000
Catholic	58.5	41.5	1304	
Muslim	47.0	53.0	385	
Pentecostal	66.1	33.9	286	
Others	66.7	33.3	90	
Marital status				
Never in union	86.2	13.8	87	0.000
Married	57.6	42.5	1981	
Cohabiting	55.3	44.7	882	
Widowed	66.7	33.3	21	
Divorced/separated	60.4	39.6	235	
Wealth index				
Poorest	57.9	42.2	688	0.987
Poorer	57.3	42.7	647	
Middle	58.6	41.4	650	
Richer	58.5	41.5	634	
Richest	57.6	42.4	587	
Age at first sex				
Below 16	54.5	45.5	646	0.000
16–17	52.2	47.8	783	
18–19	56.8	43.2	901	
20–21	64.9	35.1	419	
22 plus	68.7	31.3	457	
Contraceptive type				
No method	57.3	42.7	1792	0.526
Traditional method	61.5	38.5	161	
Modern method	58.5	41.5	1253	
Circumcision status				
No	59.4	40.6	1831	0.067
Yes	56.2	43.9	1375	
Sex partners excluding spouse				
None	58.8	41.3	2487	0.265
One	55.2	44.8	594	
Two plus	56.0	44.0	125	
	OR	Std. Err	z	*p*-value
Nos. of wives/partners	3.12	0.24	14.53	0.000
Nos. of lifetime sex partners	1.02	0.03	7.00	0.000

Results from the logistic regression model are summarized in Table 3. Regarding age, for males aged 25–29 years, the odds of fathering children with multiple partners were 2.23 times higher compared to males below 25 years other factors constant. Similarly, males aged 30–34 years (AOR = 3.71), 35–39 years (AOR = 5.30), 40–44 years (AOR = 5.79) and above 44 years (AOR = 4.43) were more likely to father children with multiple partners compared to males below 25 years. Males residing in the Western region had lower odds (AOR = 0.79) of fathering children with multiple partners compared to their counterparts in the Central region. As regards religion, Muslims (AOR = 1.35) were more likely to father children with multiple partners compared to Anglicans. Married males (AOR = 0.34) were less likely to father children with multiple women compared to those who had never been in union. Likewise, cohabiting males (AOR = 0.46) were less likely to father children with multiple women compared to those who had never been in union. Divorced or separated males (AOR = 2.14), were more likely to father children with multiple partners compared to those who had never been in union. Pertaining to age at first sex, males who first had sex aged 20–21 years (AOR = 0.54) and above 21 years (AOR = 0.44), were less likely to father children with multiple partners compared to those who first had sex below 16 years. Still, the odds of fathering children with multiple partners increased with increase in the number of wives/partners a male had (AOR = 5.60). Finally, the odds of fathering children with multiple partners increased with increase in the number of lifetime sex partners a male had (AOR = 1.01).

**Table 3:** Determinants of multiple partner fertility

Variables	AOR	95% CI	
Age (years)			
Below 25 (ref.)	1.00		
25–29	2.23**	1.58	3.13
30–34	3.71**	2.66	5.17
35–39	5.30**	3.74	7.50
40–44	5.79**	4.08	8.21
Above 44	4.43**	3.15	6.21
Region			
Central (ref.)	1.00		
Eastern	1.09	0.87	1.37
Northern	1.08	0.84	1.38
Western	0.79**	0.63	1.00
Religion			
Anglican (ref.)	1.00		
Catholic	0.89	0.74	1.07
Muslim	1.35**	1.03	1.76
Pentecostal	0.79	0.59	1.06
Others	0.72	0.43	1.21
Marital status			
Never in union (ref.)	1.00		
Married	0.34**	0.17	0.69
Cohabiting	0.46**	0.23	0.92
Widowed	1.66	0.54	5.12
Divorced/separated	2.14**	1.07	4.28
Age at first sex (years)			
Below 16 (ref.)	1.00		
16–17	1.00	0.79	1.26
18–19	0.84	0.67	1.06
20–21	0.54**	0.40	0.71
Above 21	0.44**	0.33	0.58
Nos. of wives/partners	5.60**	4.39	7.14
Nos. of lifetime sex partners	1.01**	1.01	1.02

**p ≤ 0.05, (ref.) – reference category

## Discussion

This study set out with the aim of identifying the determinants of multiple partner fertility among males in Uganda. Secondary data obtained from the Uganda Demographic and Household Survey (UDHS) of 2016 were applied to this study.

The results of this study indicated that the probability of fathering children with multiple women increased with increase in one’s age. This result was consistent with the findings of the study by Logan et al. [2] who observed a steady increase in the proportion of males fathering children with multiple women from 5% by the age of 25 to 15% by the age of 40. A possible explanation for this result may be attributed to the longer duration of time that older men have had to move from one relationship to another, compared to younger men [5]. Secondly, older men tend to have more resources than younger men [15] and they are therefore in a better position to have another child from a different relationship.

It is interesting to note that males in the Western region had lower odds of fathering children with multiple partners compared to males in the Central region. This can be attributed to the fact that the Central region is more urbanized than the Western region of Uganda. According to the 2014 National Population and Housing Census report, at least half of the twenty largest urban centers were located in Central Uganda and only three in Western Uganda [16]. Also, findings from the UDHS report by UBOS and ICF [14] indicated that 36.2% of urban males compared to 28.4% of rural males reported having sex in the past 12 months with a person who was neither their wife nor lived with them. Therefore, given that males in urban areas tended to have multiple sexual partners, this may explain the higher odds of male multiple partner fertility in the more urbanized Central region compared to the Western region.

As expected, this study demonstrated that the Muslim men had greater odds of multiple partner fertility than men belonging to the Anglican religion. This result can be attributed to the teachings in the Quran (4:1) which do not prohibit a man from having more than one wife [17]. For this reason, the men are at liberty to marry more than one wife and have children with them. Religiosity has an impact on the attitude of an individual towards different aspects of marriage such as divorce and commitment and longevity [18, 19].

The findings from the analysis of the data in this study showed that males who were cohabiting or married had lower odds of fathering children with more than one woman compared to the males who were not in union. Males who were divorced or separated had higher odds of fathering children with more than one woman compared to males who were not in union. Although people who are cohabiting are in less legalized relationships and are less bound to commit to a single relationship [20], the findings of the study could be attributed to partners in this case looking at cohabitation as a step towards getting married [21]. Therefore, males who are cohabiting may demonstrate the same level of commitment to a specific partner even though the relationship isn’t yet formalized by getting married. The males who are divorced or separated are at greater liberty to start another relationship and have children with the women in the new relationship compared to males who are not divorced.

Another important finding in the study was that the odds of male multiple partner fertility increased with decrease in age of first sex. The result in this study was in agreement with Logan et al.’s [2] study who found out that 27, 16 and 6% of males whose age of first sex was by 15, 17 and 19 respectively had fathered children with more than one woman. Early sexual debut is linked to increased risk of involvement in risky sexual behaviors such as having unprotected sex, unstable sexual partners and unwanted pregnancies [22–24]. In addition, the men who birthed their first child at an early age were less likely to be married at the time which reduced their likelihood of committing to just one woman, the mother of their first child [5]. The increased odds of multiple partner fertility among males with many wives/partners is expected since having more children is one of the reasons for males getting other wives or partners especially in developing countries [25–27]. Finally, the odds of fathering children with multiple women increased with increase in number of lifetime sex partners. This could be due to the increased tendency by males to use protection against pregnancy less reliably as their number of sexual partners increases [28]. Ashenhurst et al.’s [28] study found that people with multiple partners over time had the highest odds of reporting sex with no protection against STIs and pregnancy compared to those who had one partner over time.

The most important limitation of this study lies in the fact that data in the UDHS is dependent on the participant’s responses which may be affected by recall, reporter or response bias. Also, the men may not be in the know of the actual number of children they have fathered outside their current union and with how many partners [2]. These affect the accuracy of the information given. Secondly, this was a cross-sectional study which limits the research to description of the association and not the cause-effect relationship between male multiple partner fertility and identified factors. Thirdly, since secondary data were used, some key variables were left out of the study given that they weren’t captured. These included marital status of one’s parents at their birth, family structure, number of siblings and education level of their parents etc. [5].

## Conclusions

This paper aimed to identify the determinants of multiple partner fertility among males in Uganda. The evidence from this study implied that current age, number of wives or partners and number of lifetime sex partners were the strongest predictors of multiple partner fertility among males in Uganda. Other predictors were age at first sex, region, religion and marital status although not all categories were significant. The findings have important implications on the development of Uganda, a country with a high proportion of young people. A sizeable proportion (42%) of fathers had children with more than one woman. Multiple partner fertility has a significant effect on the health and wellbeing of the children, particularly those from the previous partner or who do not live with their fathers. These children tend to have less resources, guidance, support and time with both biological parents [7]. This greatly affects their access to basic services (such as education and health) and overall productivity in life. There is a need to come up with policies and programs aimed at increasing the age at first sex so as to reduce the likelihood of MPF. These can include educating young people about the negative outcomes of having sex at an early age. Government and other stakeholders such as cultural and religious institutions should sensitize and educate the masses on the negative outcomes of having children with multiple partners and promote fidelity for those in marriage. There is also need to improve on access to and utilization of modern contraceptive methods as a preventive mechanism against unintended pregnancies.

## Data Availability

The dataset used for this study is publicly available upon formal request from the DHS program website. https://dhsprogram.com/data/dataset/Uganda_Standard-DHS_2016.cfm?flag=0

## Abbreviations

AOR: Adjusted Odds Ratio
EA: Enumeration Area
HIV: Human Immunodeficiency Virus
ICF: ICF (originally, *Inner City Fund*)
MPF: Multiple Partner Fertility
NPHC: National Population and Housing Census
UBOS: Uganda Bureau of Statistics
UDHS: Uganda Demographic and Health Survey
UNAIDS: Joint United Nations Programme on HIV/AIDS
USA: United States of America
